# *Fasciola hepatica* in Some Buffaloes and Cattle by PCR and Microscopy

**DOI:** 10.1155/2014/462084

**Published:** 2014-11-13

**Authors:** Sultan Ayaz, Riaz Ullah, Naser M. AbdEl-Salam, Sumiara Shams, Sadaf Niaz

**Affiliations:** ^1^College of Veterinary Sciences and Animal Husbandry, Abdul Wali Khan University Mardan, Khyber Pakhtunkhwa 23200, Pakistan; ^2^Department of Chemistry, Government College Ara Khel, FR Kohat, Khyber Pakhtunkhwa 26000, Pakistan; ^3^Riyadh Community College, King Saud University, Riyadh 11437, Saudi Arabia; ^4^Department of Zoology, Abdul Wali Khan University Mardan, Khyber Pakhtunkhwa 23200, Pakistan

## Abstract

Fasciolosis is the burning problem of the livestock rearing community having huge morbidity, mortality, and economic losses to livestock industries in our country Pakistan. The faecal and liver biopsy samplings were examined by polymerase chain reaction (PCR) and microscopy technique during the entire study. A total of 307 samples including 149 samples from Karak and 158 samples from Kohat abattoirs were examined by PCR method and overall prevalence of fasciolosis was 5.86% (18/307), amongst theses 8.05% (12/149) in liver biopsy and 3.79% (6/158) in feacal samples of cattle and Buffaloes were recorded. Similarly the microscopy based detection was 3.58% (11/307) including 4.61% (7/149) in liver biopsy and 2.5% (4/158) in faecal samples accordingly. Furthermore the areawise prevalence of fasciolosis in abattoirs by PCR method was found to be 7.59% (12/158) in Kohat and 4.02% (6/149) in Karak. A 618 pb DNA was amplified in 2% agarose gel electrophoreses. It is concluded from the study that prevalence of fasciolosis was higher in abattoir of district Kohat and PCR was a more sensitive method of diagnosis than microscopy.

## 1. Introduction

Fasciolosis in important food born and water born parasitic zoonosis caused by liver fluke of the genus* Fasciola* [1, 2], the* F. hepatica,* is cosmopolitan in distribution, with high frequency in tropical area [3, 4].* Fasciola* spp. may reach the size of 25–30 mm in length and 8 to 15 mm width. It has leaf shaped Structure [5].* Fasciola hepatica* has an interior and posterior sucker for attachment to host body [6].* Fasciola hepatica* completed its entire life cycle in two host cattle, a definitive host, and the snail, an intermediate host, while the human is an accidental host [1, 7], which causes disease mostly in ruminants, especially in cattle, buffaloes, sheep, goats, and cow. It may however affect human [8].

These parasites inhabit the hepatobiliary system of the effected animal and rarely can be found in ectopic sites within the host body [9]. Once the parasites eggs are ingested by the cattle by the occasional drinking or grazing, then the parasites migrate through the liver parenchyma to reach the bile duct. The diagnosis of fasciolosis in ruminant caused by* Fasciola* spp. has been made solely by the detection of* Fasciola* eggs in the faeces of infected animal [10].

The worldwide losses in animal productivity due to fasciolosis were estimated as US $200 million per annum to rural agricultural communities and commercial producers with over 600 million animals infected. In developed counties, the incidence of* F. hepatica* can reach up to 77%. In tropical countries, fasciolosis is considered the single most important helminthes infection of cattle, with reported prevalence of 30–90%. In domestic ruminants, adverse effects of acute or chronic fasciolosis include decreased meat and milk production, decreased fertility, and increased veterinary costs [11–13].

Fasciolosis is one of the big and most important worldwide problems mainly due to mortality of animals, cost of diagnosis, and treatment of condemned liver and it reduces milk and meat production, fertility disorder, and drug resistance against fasciolosis [14]. The present research project was designed to carry out the PCR base prevalence of fasciolosis in cattle and buffaloes in abattoir of district Karak and Kohat.

## 2. Materials and Methods

### 2.1. Samples Collection

A total of 307 samples including 158 faecal samples and 149 liver samples were collected from the abattoir of district Kohat and Karak Khyber Pakhtunkhwa from the cattle having different sex and age. Faeces samples were directly collected from the rectum of the cattle in polythene bags which is duly labeled according to sex, age, date, and abattoir from which the samples were collected and similarly the liver samples were collected after slaughtering of those animal which are clinically suspected (having blister or swelling on the liver surfaces) where the faecal samples of the animals were collected. The targeted swelling parts of the liver were incised with scalpel and put in a sterilized bottle duly labeled with date, spp., sex, and breed of the animal. The collected samples were placed in ice jar and were immediately transported to the Virology and Molecular Parasitology Laboratory of the Zoology Department, Kohat University of Science and Technology, Kohat.

### 2.2. Microscopy

Thick and thin smears were prepared from the faecal and liver biopsy including bile duct material. Both of faeces and liver biopsy were mixed with buffer saline and a drop of 20 *μ*L was placed on the slides and dried and were puts a drop of immersion oil and then observed under the direct microscopy of 10x, 40x, and 100x. The images were compared with the standard morphological size.

### 2.3. DNA Extraction

The samples were subjected to DNA extraction by using GF-1 kit (vivantis) as per the manufacturer protocol (Sultan Ayaz PhD thesis, 2009 HEC Pakistan panel). A 200 *μ*L liver biopsy as well as the faecal pellet samples in Eppendorf tube was mixed with 50 *μ*L of proteinase K and 200 *μ*L of buffer VL. They were mixed well with the help of vertex and then were incubated at 65°C for 10 min in hot plates. The columns were centrifuged at 6000 rpm for 1 min and the flow through was discarded; then 200 *μ*L of wash buffer was added and centrifuged at 6000 rpm for 1 min and again the supernatant was discarded. Similarly 200 *μ*L of wash buffer 2 was added and centrifuged at 6000 rpm for 1 min and supernatant was discarded. Then the columns were transferred to new tubes and 30 *μ*L of elution buffer was added and placed for 2 min at room temperature. After that centrifugation was at 6000 rpm for 1 min and was mixed with 30 *μ*L of deionized water and stored at −80°C for further process.

### 2.4. DNA Amplification (PCR)

The DNA was amplified through polymerase chain reaction (PCR) using primer specific for* Fasciola hepatica* (Figure 2). The primer added for* F. hepatica* was -F, 5′-AGTGATTACCCGCTGAACT-3′, and R, 3′-CTGAGAAAGTGCACTGACAA-5′ [13]. The specific amplified product was compared with 100 bp DNA ladder marker (Fermentas, USA). The parasitic DNA was recognized.

The target DNA was amplified in 20 *μ*L reaction mixture containing 10x PCR buffer 2 *μ*M, 1 *μ*M deoxynucleoside triphosphate (500 *μ*M), 2.4 *μ*M MgCl_2_ (25 *μ*m) 1 *μ*M primers (10 pmol), target DNA 5 *μ*L, and 0.3 unit of* Taq* DNA polymerase (5 u/*μ*L); add deionized water up to 20 *μ*L. Denaturing of DNA amplification was done at temperature (92°C for 3 min, 25 cycles), (92°C for 40 sec), (50°C for 40 sec) and (72°C for 60 sec). In the last stage extension at 72°C for 7 min and hold at 4°C for unlimited time (Table 1), the designed program was saved.

### 2.5. Gel Electrophoresis

In gel electrophoresis, 2 g of Agarose was added in 100 mL of TBE buffer and placed in oven for 2 minutes at 100°C. Then removed this mixture and cool down it up to 45°C after then added 20 *μ*L of ethidium bromide. The gel was poured into gel tray and combs were fixed. Combs were removed after gel was formed. So by this way, the Gel tray was placed in gel tank containing 1000 mL 0.5x TBE buffer. 10 *μ*L Of PCR product was mixed with 5 *μ*L of bromophenol blue dye of each sample was loaded in the wells and 15 *μ*L of DNA ladder (100 bp) was loaded in the separate well. The positive and negative control was run parallel with the samples. The gel was run for 25 min at voltage of 130 volts and 500 ampere current. Gel was then examined by UV transilluminator. A photo was cached and saved in a record.

## 3. Results and Discussion

**Figure 1:**
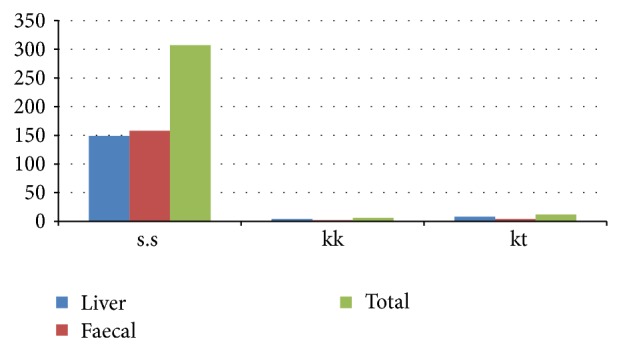
Prevalence of fasciolosis cattle and buffaloes by using PCR and microscopy methods in Karak and Kohat, Pakistan.

**Figure 2:**
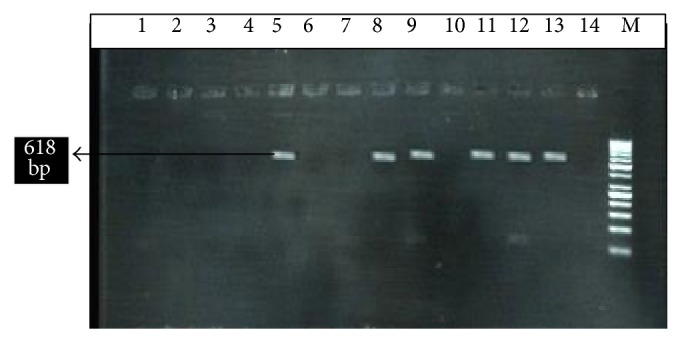
M; 100 bp DNA ladder, lane 5 is positive control, while lane 14 is negative control. Lanes 13, 12, 11, 9, and 8 are positive and the other lanes 1, 2, 3, 4, 6, 7, and 10 are negative. The amplified DNA band showing 618 bp.

Fasciolosis is a very serious disease, having huge economic losses of the cattle and industries in terms of meat, milk, and leather in our country. In the current study a total of 307 samples were examined, which included 149 samples from Karak and 158 samples from Kohat of the cattle and buffalos of Khyber Pakhtunkhwa. By examination it is shown that the overall prevalence of fasciolosis was 5.86% (18/307), amongst these 4.02% (6/149) in the district Karak and 7.5% (12/158) in the district Kohat. Furthermore the prevalence of Fasciolosis in cows was 3.07% (2/65) and 4.76% (4/84) in the Buffaloes of the district Karak was recorded while 5.19% (4/77) in the Cow and 9.87% (8/81) in the Buffaloes of district Kohat of the Khyber Pakhtunkhwa were recorded. The prevalence of fasciolosis was higher in district Kohat as compared to district Karak. Statistical analysis revealed the significant difference *P* < 0.05 when the data was interpreted (Tables 2, 3, and 4) (Figure 1).

In the present study,* F. hepatica* was found in the fecal sample and liver biopsy sample of cows and buffaloes in the abattoir of the district Karak and Kohat of the Khyber Pakhtunkhwa province of Pakistan.

One of the studies revealed that it was the disease of domesticated animals in Sindh province that causes heavy infection of* F. hepatica*. Moreover,* F. gigantica *was reported at high altitudes in Khyber Pakhtunkhwa province, whereas* F. hepatica* occurred in deltaic region of Punjab and Sindh provinces, Pakistan. Similar, findings were previously reported In Faisalabad (central Punjab) [15]; overall prevalence of fasciolosis was found to be 17.55%, of which* F. hepatica* was 5.7%. However mixed infection was revealed in 2.02% animals [16].

*Fasciola hepatica* was the dominant fluke species in the animals [17]. This may be associated with the existence of favorable ecological biotopes for* Lymnaea truncatula*, the recognized intermediate host of* F. hepatica* in Ethiopia [18].

The worldwide losses in animal productivity due to fasciolosis were estimated at US $200 million per annum to rural agricultural communities and commercial producers [19], with over 600 million animals infected [20]. In developed counties, the incidence of* F. hepatica* can reach up to 77%. In tropical countries, fasciolosis is considered the single most important helminthes infection of cattle, with reported prevalence of 30–90% [21]. This study coincided partially with our study in cattle and buffaloes.

Similar study from Northern Iran (Tonekabon) indicates a 15% prevalence of fasciolosis in buffaloes and 4.6% in cattle [22]. Fasciolosis is now recognized as an emerging food born zoonosis in many parts of the world and world health organization has also included human fasciolosis on its list [23].

## Figures and Tables

**Table 1:** Settings of PCR cycle for *F. hepatica*.

Stage	Cycle	Step	Temperature	Time
1	1	1	92°C	3:00 min

2	25	1	92°C	40 sec
		2	50°C	40 sec
		3	72°C	1:00 min

3	1	1	92°C	7:00 min
		2	92°C	Hold

**Table 2:** Prevalence of fasciolosis in cattle and buffaloes in district abattoirs of Karak and Kohat by using PCR and microscopy methods.

Sample	Spp. cattle + buffaloes from Karak + Kohat	Karak PCR positive (cattle + buffaloes) %	Kohat PCR positive (cattle + buffaloes) %	Prevalence (PCR) %	Microscopy prevalence %
Liver sample	70 + 79 = 149	4 5.71% (4/70)	8 10.12% (8/79)	4 + 8 = 12 8.05% (12/149)	7/149 4.6%

Faecal sample	79 + 79 = 158	2 2.53% (2/79)	4 5.06% (4/79)	2 + 4 = 6 3.79% (6/158)	*4/158* *2.5% *

G. total	149 + 148 = 307	4 + 2 = 6 4.02% (6/149)	8 + 4 = 12 7.59% (12/158)	6 + 12 = 18 5.86% (18/307)	11/307 3.58%

**Table 3:** PCR based detection of fasciolosis in the district abattoir of Kohat.

Sample	Cow		Buffalo		Total sample		Prevalence %		Total prevalence %	Other findings
	Positive sample	Negative sample	Positive sample	Negative sample	Cow	Buffaloes	Cow	Buffalo		
Faecal sample	1	38	3	37	39	40	2.56% (1/39)	7.5% (3/40)	5.06% (4/79)	*E. histolytica* and *G. lamblia* cryptosporidium

Liver sample	3	35	5	36	38	41	8.33% (3/38)	12.19% (5/41)	10.12% (8/79)	No other findings

G. total	4	73	8	76	77	81	5.19% (4/77)	9.8% (8/81)	7.59% (12/158)	*E. histolytica * and *G. lamblia * cryptosporidium

**Table 4:** PCR based detection of fasciolosis in the district abattoir of Karak.

Sample	Cow		Buffalo		Total sample		Prevalence %		Total prevalence %	Other findings
	Positive sample	Negative sample	Positive sample	Negative sample	Cow	Buffaloes	Cow	Buffalo		
Faecal sample	1	39	1	38	40	39	2.5% (1/40)	2.56% (1/39)	2.53% (2/79)	*E. histolytica * and *G. lamblia* cryptosporidium

Liver sample	1	24	3	42	25	45	4% (1/25)	6.66% (3/45)	5.71% (4/70)	No other findings

G. total	2	63	4	80	65	84	3.07% (2/65)	4.76% (4/84)	4.02% (6/149)	*E. histolytica* and *G. lamblia * cryptosporidium
